# Factors associated with breastfeeding at six months postpartum in a group of Australian women

**DOI:** 10.1186/1746-4358-1-18

**Published:** 2006-10-12

**Authors:** Della A Forster, Helen L McLachlan, Judith Lumley

**Affiliations:** 1 Mother and Child Health Research, La Trobe University, 251 Faraday St, Carlton 3053, Australia; 2 Clinical School of Midwifery and Neonatal Nursing Studies, La Trobe University, 251 Faraday St, Carlton 3053, Australia

## Abstract

**Background:**

Despite high levels of breastfeeding initiation in Australia, only 47 percent of women are breastfeeding (exclusively or partially) six months later, with marked differences between social groups. It is important to identify women who are at increased risk of early cessation of breastfeeding.

**Methods:**

Data from the three arms of a randomised controlled trial were pooled and analysed as a cohort using logistic regression to identify which factors predicted women continuing to feed any breast milk at six months postpartum. The original trial included 981 primiparous women attending a public, tertiary, women's hospital in Melbourne, Australia in 1999–2001. The trial evaluated the effect of two mid-pregnancy educational interventions on breastfeeding initiation and duration. In the 889 women with six month outcomes available, neither intervention increased breastfeeding initiation nor duration compared to standard care. Independent variables were included in the predictive model based on the literature and discussion with peers and were each tested individually against the dependent variable (any breastfeeding at six months).

**Results:**

Thirty-three independent variables of interest were identified, of which 25 qualified for inclusion in the preliminary regression model; 764 observations had complete data available. Factors remaining in the final model that were positively associated with breastfeeding any breast milk at six months were: a very strong desire to breastfeed; having been breastfed oneself as a baby; being born in an Asian country; and older maternal age. There was an increasing association with increasing age. Factors negatively associated with feeding any breast milk at six months were: a woman having no intention to breastfeed six months or more; smoking 20 or more cigarettes per day pre-pregnancy; not attending childbirth education; maternal obesity; having self-reported depression in the six months after birth; and the baby receiving infant formula while in hospital.

**Conclusion:**

In addition to the factors commonly reported as being associated with breastfeeding in previous work, this study found a negative association between breastfeeding outcomes and giving babies infant formula in hospital, a high maternal body mass index, and self-reported maternal depression or anxiety in the six months after the baby was born. Interventions that seek to increase breastfeeding should consider focusing on women who wish to breastfeed but are at high risk of early discontinuation.

## Background

There is strong evidence to support the World Health Organization (WHO) recommendation for women to breastfeed infants exclusively for the first six months of life, with continued breastfeeding in combination with the gradual introduction of other forms of nutrition beyond that time and into the second year [1]. Most infants in Australia, as in many high-income countries, are not exclusively breastfed for the first six months. Although 80–90% of Australian women initiate breastfeeding, fewer than half are giving their infants any breast milk by six months [2,3], with marked differences by socio-economic group [2].

This paper focuses on the factors affecting breastfeeding *duration*; those associated only with the *initiation *of breastfeeding are not discussed. The literature regarding factors associated with breastfeeding duration is extensive; those summarised here were identified as part of a substantial literature review [4] where papers were selected purposively based on study type. Priority was given to: studies where data had been systematically collected prospectively as part of cohort studies or randomised controlled trials; recent significant articles of relevant subject areas, for example where a substantial literature review had been undertaken; and studies that considered confounding factors by conducting multivariate analysis to adjust for confounders or interactions.

Given the breadth of the literature, Table 1 has been developed to present a summary of the factors associated with duration of breastfeeding, stratified by category. Major categories considered are: maternal factors; hospital practices and obstetric factors; and other potential influences. Factors which are consistently reported as being positively associated with duration of breastfeeding are an intention to breastfeed [5-15]; earlier timing of the decision to breastfeed [7,9,16-18]; increasing maternal age [8,9,11-13,15,18-23]; higher maternal education [7,9,11,14,19,21,24-26]; not smoking [19,21,23,27-30], or smoking less [25]; and being married [21] or not being single [22].

**Table 1:** Factors associated with the duration of breastfeeding, stratified by consistency of reports

**Factors**	**Consistent associations reported in the literature**		**Associations reported in the literature but not found consistently**	
	**Positive association – breastfeeding duration**	**Negative or *no *association – breastfeeding duration**	**Positive association – breastfeeding duration**	**Negative or *no *association – breastfeeding duration**

**Maternal & social factors**	Intention to breastfeed [5, 6-15]	Higher body mass index [27, 46, 47]	Having a previous child/children [21, 23]	Previous infant feeding method *(no association) [9]*
	
	Earlier timing of decision to breastfeed [7, 9, 16-18]		Having a previous baby increased *planned *duration [6]	Breastfeeding confidence *(no association) [7]*
	Increasing age [8, 9, 11-13, 15, 18-23]		The better the previous breastfeeding experience, the more positive association with subsequent breastfeeding duration [14, 23]	Lower income [48]
	
	Higher education [7, 9, 11, 14, 19, 21, 24-26]		Breastfeeding confidence [6, 8, 10, 14, 22, 32, 49]	A view that the feeding method makes no difference to the baby's health [42]
	
	Not smoking [19, 21, 23, 27-30] or smoking less [25]		Higher social class [50, 51]	More positive attitude to bottle feeding [11]
	
	Being married [21] or not being single [22]		Higher income [20]	Employment plans *(no association) *[9, 11, 12]
	
			Higher socioeconomic status [2]	Returning to work [22]
	
			Maternal attitude to infant feeding [24] or negative attitude to formula feeding [7]	Parity *(no association) *[20]
	
			Having been breastfed oneself [42]	Perception of lack of support for breastfeeding [6]
	
			Attendance at childbirth education classes [9]	
	
			Partner's perceived preference for breastfeeding [12, 22, 42, 52]	
	
			Breastfeeding knowledge [53]	

**Hospital practices & obstetric factors**		Early postnatal discharge *(no association) *[5, 23, 55, 56]	Earlier breastfeeding initiation [7,10, 18, 32, 57, 58]	Caesarean section [9]
	
		Early postnatal discharge [59]	Rooming-in [12, 32, 60]	Birth type *(no association) *[7, 61]
	
		Commercial discharge packs *(but effect more on exclusivity) *[62]	Early skin-to-skin contact [63, 64]	Early infant to breast contact *(no association) *[12]
	
			Breastfeeding encouragement from a health professional [22]	Use of formula during the postnatal hospital stay [26, 65] (*but not associated in earlier meta-analysis [64])*

**Other potential influences**		Introduction of solids with continued breastfeeding *(no association) *[66]	Mastitis [15]	Breastfeeding problems such as sore and cracked nipples *(no association) *[7]
	
		Introduction of formula [10, 15, 58, 66]		Early breastfeeding problems [22]
	
				Inverted nipples [15]
	
			Infant birth weight >2500 g [21]	Lower infant birth weight [23]
	
				Infant birth weight *(no association) *[9]
	
				Male infant [12]
	
				Admission to special care nursery [12]
	
				Use of dummies (pacifiers) [15, 25, 26]
	
				Use of dummies (pacifiers) *(no association) *[67]

Some factors were so inconsistent in their reported association with breastfeeding that they were unable to be categorised for consistency of association. Ethnicity has had an association with breastfeeding duration in some studies from multicultural communities, though the direction of the association is inconsistent [19,22,31,32]. For example, migrant women from some groups may be less likely to breastfeed in a new country [31,33,34], whereas women from other cultural backgrounds may have higher rates of breastfeeding than non-migrant women [31,34]. With some factors such as maternal employment, it is difficult to compare the findings between studies, given that different countries have differing maternity leave entitlements and structures.

Whilst there are trends across countries and cultural groups regarding the factors which influence or predict breastfeeding outcomes, it seems clear from the literature that there are many factors that influence breastfeeding, and different factors will be in play depending on individual circumstances. There are however, certain groups for whom the evidence is consistent, regardless of culture and ethnicity, and for whom the risk of early breastfeeding cessation (or non-initiation of breastfeeding) is higher, such as younger women who have less education and who are single.

The aim of this paper is to use combined data from a randomised controlled trial to describe and present the factors which predicted or were associated with women continuing to breastfeed any breast milk until at least the time of a telephone interview six months postpartum. Factors that are associated with six month breastfeeding outcomes and appear amenable to change could be used to plan interventions aimed at increasing the proportion of women breastfeeding to six months and beyond.

Ethics approval was obtained from The Royal Women's Hospital and La Trobe University Human Research Ethics Committees.

## Methods

The current paper uses data collected in a randomised controlled trial designed to test the effect of two different educational interventions provided in mid-pregnancy on the initiation and duration of breastfeeding (the ABFAB trial) [35]. Participants were randomly allocated to a control group or one of two small-group interventions: a previously designed and tested tool to teach practical aspects of breastfeeding [36] or an exploration of family attitudes to breastfeeding. All eligible women booking to have a baby at the Royal Women's Hospital (RWH) (Melbourne) between May 1999 and August 2001 were approached to participate. Inclusion criteria were: women booking as public patients; having a first child; between 16 and 24 weeks pregnant at the time of recruitment; able to speak, read and write in English. Exclusion criteria included physical problems that prevented breastfeeding or choosing birth centre or private obstetric care. In Australia, women who book for either of these two models of pregnancy care are more likely to initiate and continue breastfeeding.

Demographic data and information on women's breastfeeding intentions were collected at recruitment by self-administered questionnaire, prior to randomisation. Primary and secondary outcome data were collected by interview in hospital after the birth (or by telephone if the woman was already discharged) and by telephone interview at six months, using structured questionnaires. Medical/obstetric hospital data were obtained electronically from the hospital data system after each woman had her baby.

The sample size for the original trial had the power to identify an increase in breastfeeding at discharge for primiparae who were public patients from 75 % (audit data 1998) to 85 % (estimated as the proportion in private patients), with 95 % confidence and 80 % power. Two hundred and seventy women per group were required for this, and allowing for 20 % loss to follow-up, the required numbers were 324 per group, or 972 in total [37]. This sample size also had the power to identify an increase in breastfeeding at six months from 38 % (estimated from unpublished 1995 Victorian Maternal and Child Health data as likely for RWH primiparae) to 52 % in either intervention group compared with the control group. Of the 981 women recruited to the trial, outcome data were available for 889 women at six months postpartum. Neither intervention increased breastfeeding initiation or duration compared to standard care. Overall, 96% of women were feeding at least some breast milk at two to four days after the birth and 53% were continuing to feed at least some breast milk at the time of the six month telephone interview [35]. With respect to the sample size for this analysis we used the 'rule of ten'; that is, that at least ten 'cases' are required for each variable in the model [38].

Given the strength and consistency of the association between infant feeding intention and breastfeeding outcomes, data on feeding intention were collected at several time points. When asked at recruitment how they planned to feed their baby, 26% of women responded that they planned to breastfeed for six months or more. When asked after the birth if, and how long they planned to breastfeed, 63% of women said they planned to breastfeed for six months or more. When asked at the six month interview what their original intentions were, an even higher proportion reported that they had originally intended to breastfeed for six months or longer (73%). In the regression model the first two variables were combined to take into account the change of intention, to become a single variable with four categories: 'Intended to breastfeed six months or more at recruitment and in hospital interview'; 'Did not intend to breastfeed six months at recruitment but DID at in hospital interview'; 'Intended to breastfeed six months at recruitment but NOT at in hospital interview'; and 'Never intended to breastfeed six months or more'.

Outcome data were combined for use in a logistic regression model to explore which factors predicted women continuing to breastfeed at six months postpartum. *Any *breastfeeding at six months was the outcome of interest (dependent variable). Independent variables were included in the model based on the literature and discussion with peers, and were each tested individually against the dependent variable. Most of the data collected were categorical, and are presented in those categories, for example smoking pre-pregnancy, where women ticked the response category which was correct for them. In some cases responses were collected in more than one question, but have been collapsed into one outcome variable to reflect a continuum. An example is education, which was collected separately as secondary and tertiary education levels, but which was combined to become one variable with three categories: did not complete secondary education; completed secondary education (but not tertiary); and completed tertiary education. Maternal body mass index (BMI) was calculated as weight in kilograms divided by height in metres squared, and the categories used were: underweight (BMI < 20); normal weight (BMI 20–25); overweight (BMI >25 and < 30); and obese (BMI ≥ 30).

Logistic regression was used for binary or categorical variables, t-tests for continuous variables that were normally distributed and the Mann-Whitney test for continuous variables which were not normally distributed. Variables were included in the preliminary regression model if the p-value of the Wald statistic was ≤ 0.2, or if there was an *a priori *reason for their inclusion. Once the variables were assigned to the preliminary model, a decision was made regarding missing values. Where possible, all observations were retained. Categorical variables in which the issue of being missing may in itself have had some influence on the outcome had a code added to categorise 'missing', so all observations could be included in the model. In other variables where there was no pattern to 'missingness', observations that had missing data were dropped from the dataset.

All variables chosen for inclusion in the preliminary model were entered into the regression model, and variables were eliminated one at a time with subsequent regressions. Only variables with a Wald statistic p-value of = 0.05 were retained in the model [38]. The likelihood ratio test was used to test each subsequent model with the previous one, to ensure that the newer, simpler model did not differ significantly from the previous model except for the removal of the relevant variable. The process was repeated until only significant variables remained.

A range of tests and checks were conducted prior to considering the model final: continuous variables were checked to ensure that the relationship between the continuous variable and outcome was a linear association; all covariates eliminated following the original univariate analysis were added back into the model one at a time to check that none had become significant given the reduced model (any significant variables would have been retained, but there were none in which this was the case); and the model was tested for interactions and confounders as well as goodness of fit. As a final step in the modelling process, individual covariate patterns were examined to check for outliers which may have exerted extreme influence or leverage on the model. STATA [39] was used for data analysis.

## Results

Some demographic data are presented in Table 2 to demonstrate that the women who were available for data collection at six months (n = 889) were very similar to those who were originally recruited (n = 981). Table 3 shows the characteristics of sample for each independent variable included in the final model (n = 764).

**Table 2:** Comparison of demographic data at recruitment (n = 981) and those remaining in sample at six months (n = 889)

**Demographic characteristics**	**n**	***%*** ***(of 981)***	**n**	***%*** ***(of 889)***
Lives with husband/partner	864	*88.0*	788	*88.6*
Completed secondary school	734	*74.9*	670	*75.4*
Pension/benefit main family income	122	*12.4*	101	*11.4*
Smoked prior to pregnancy	362	*36.9*	324	*36.4*
English first language	813	*82.9*	744	*83.4*
Age at recruitment, years (mean, sd)	28.3	*(5.7)*	28.3	*(5.6)*

**Table 3:** Characteristics of sample for each independent variable (n = 764)

**Factor**	**No**	***%***
**Had *antenatal *intention to breastfeed 6 months or more**	203	*26.6*
**Had *postnatal *intention to breastfeed 6 months or more**	511	*66.9*
**Desire to breastfeed**		
Very strong desire to breastfeed	595	*77.9*
Desire to breastfeed goes up and down	120	*15.7*
Sometimes think bottle (formula) feeding preferable	46	*6.0*
I think bottle (formula) feeding is preferable	3	*0.4*
**Confidence in breastfeeding ability**		
Feel confident in ability to breastfeed baby	360	*47.1*
Breastfeeding difficult now but hope will get easier	368	*48.1*
Not feeling confident in ability to breastfeed baby	36	*4.7*
**Partner's view of breastfeeding**		
My partner would prefer me to breastfeed	498	*65.2*
My partner does not mind how I feed the baby	22	*2.9*
My partner is supportive either way	232	*30.4*
My partner would prefer me to formula feed	8	*1.2*
Not sure	4	*0.5*
**Family's view of breastfeeding**		
My family would prefer me to breastfeed	396	*51.8*
My family would prefer me to formula feed	30	*3.9*
My family is supportive either way	311	*40.7*
Not sure	27	*3.5*
**Was breastfed as a baby**		
Breastfed as a baby	538	*70.4*
Not breastfed as baby	190	*24.9*
Own breastfeeding history not known	36	*4.7*
**Smoking pre-pregnancy (number per day)**		
None	487	*63.7*
1–9	110	*14.4*
10–19	100	*13.1*
20–29	57	*7.5*
30–39	4	*0.5*
> 40	2	*0.3*
Did not answer question	4	*0.5*
**Maternal body mass index**		
Underweight (< 20)	105	*13.7*
Normal weight (20–25)	477	*62.4*
Overweight (> 25 and < 30)	104	*13.6*
Obese (>= 30)	78	*10.2*
**Baby admitted to special care**	81	*10.6*
**Gestation**	702	*91.9*
**Breastfed within 1 hour of birth**	426	*55.8*
**Received formula in hospital**	217	*28.4*
**Completed secondary education**	589	*77.1*
**Tertiary education**		
Not comp degree/diploma	343	*44.9*
Completing degree/diploma	58	*7.6*
Completed degree/diploma	363	*47.5*
**Family income (AUD)**		
<$20,000	102	*13.4*
$20–30,000	87	*11.4*
$30–40,000	116	*15.2*
$40–50,000	84	*11.0*
> $50,000	331	*43.3*
Income category not completed	44	*5.8*
**Region of birth**		
Australia	535	*70.0*
Asia	85	*11.1*
Other	144	*18.9*
**Employed/studying at 6 months postpartum**	229	*30.0*
**Attended childbirth education **	619	*81.0*
**Reported breastfeeding problems by day 2–4**	455	*59.6*
Rated midwife home visit* extremely helpful for breastfeeding issues		
Extremely helpful	140	*18.3*
Very helpful	301	*39.4*
A little helpful	129	*16.9*
Not helpful	53	*6.9*
Did not discuss/did not have home visit	141	*18.5*
**Attended breastfeeding clinic breastfeeding clinic in first few weeks**		
Had breastfeeding problems but did not attend clinic	354	*46.3*
Had breastfeeding problems, did attend clinic	142	*18.6*
Had no breastfeeding problems	162	*60.5*
**Anxiety or depression problem in first 6 months (self-report) **	281	*36.8*
**Relationship problems at all in first 6 months **	101	*13.2*
**Age at recruitment (mean, sd) **	28.6	*(5.57)*

* In Victoria, Australia, all women receiving public maternity care are offered at least one midwife home visit postpartum, in the first few days after discharge from hospital.

Thirty-three covariates of interest were identified, and tested initially at the univariate level. Of these, 25 had a p-value of ≤ 0.2 and were included in the preliminary model. Three variables in the preliminary model, smoking, income and BMI had missing values recoded as 'missing'. Observations were dropped from the dataset where values were missing for a further 15 variables, where 'missingness' was not considered to be an issue and where there was no pattern to 'missingness', leaving data from 86% (764/889) of the original sample of women available for inclusion in the model.

Variables that were identified as potentially associated with continuing to feed any breast milk by six months but were not significant at the univariate level were gender of the baby; weight of the baby; marital status; type of birth; having at least one episode of mastitis; self-reported maternal physical health problems since the birth; rating of nipple pain; and blood loss. When added back into the final model one at a time, none of these variables became significant. These variables are not included in a table. All other variables tested at the univariate level were included in the preliminary model.

The final model is presented in Table 4, with both unadjusted and adjusted odds ratios and confidence intervals. All covariates that were initially entered into the preliminary model are included in the table. Only those that remained in the final model have adjusted odds ratios presented. Numbers and proportions presented refer to the numbers included in the final regression model (n = 764); the numbers are the number of women still breastfeeding in any category and the denominator for the proportion is the total number of women in that category. For example, 404 women replied they were not confident about breastfeeding, and of those 202 (50%) were breastfeeding at six months. The odds ratio for age (and the corresponding confidence interval) has been adjusted to make more sense clinically, expressing the association with breastfeeding for each five year increase in age. This was done by multiplying the coefficient for age by five, prior to exponentiating it to obtain the odds ratio.

**Table 4:** Regression analysis – associations with feeding any breast milk at six months

**Factor**	N *	% **	**Odds Ratio**	**(95% Confidence Interval)**	Adj*** Odds Ratio	**(95% Confidence Interval)**
**Intention to breastfeed 6 months or more (asked at recruitment and 2–4 days after birth)**						
"Yes" at recruitment AND in hospital interview (ref)	107	*68.6*	1		1	
"No" at recruitment but "Yes" at in hospital interview	205	*57.8*	0.63	(0.42, 0.93)	0.69	(0.44, 1.07)
"Yes" at recruitment but "No" at in hospital interview	27	*57.5*	0.62	(0.32, 1.21)	0.84	(0.39, 1.81)
Never intended to breastfeed 6 months or more	79	*38.4*	0.28	(0.18, 0.44)	0.41	(0.25, 0.67)
**Desire to breastfeed**						
Other than very strong (ref)	54	*32.0*	1		1	
Very strong	364	*61.2*	3.30	(2.34, 4.64)	2.18	(1.45, 3.29)
**Confidence in breastfeeding ability**						
Other than confident (ref)	202	*50.0*	1		n/s	
Feels confident	216	*60.0*	1.53	(1.15, 2.02)	n/s	
**Partner's view of breastfeeding**						
Other than prefer breastfeed (ref)	132	*50.8*	1		n/s	
Prefers me to breastfeed	283	*56.8*	1.52	(1.15, 2.02)	n/s	
**Family's view of breastfeeding**						
Other than prefer breastfeed (ref)	180	*48.9*	1		n/s	
Prefer me to breastfeed	238	*60.1*	1.95	(1.49, 2.55)	n/s	
**Was breastfed as a baby**						
Not breastfed as baby (ref)	83	*43.7*	1		1	
Breastfed as a baby	318	*59.1*	1.9	(1.33, 2.60)	1.73	(1.19, 2.54)
Breastfeeding history unknown	17	*47.2*	1.15	(0.56, 2.36)	1.72	(0.74, 4.03)
**Smoked pre-pregnancy**						
No (ref)	291	*59.8*	1		1	
1–19/day	101	*48.1*	0.62	(0.45, 0.86)	1.04	(0.70, 1.52)
20 or more/day	24	*38.1*	0.41	(0.24, 0.71)	0.47	(0.26, 0.86)
Did not answer question	2	*50.0*	0.67	(0.94, 4.82)	1.59	(0.19, 13.40)
**Maternal body mass index**						
Normal weight (20–25) (ref)	272	*57.0*	1		1	
Underweight (< 20)	63	*60.0*	1.16	(0.76, 1.76)	1.15	(0.70, 1.88)
Overweight (> 25 and < 30)	54	*51.9*	0.71	(0.47, 1.06)	0.70	(0.43, 1.12)
Obese (>= 30)	29	*37.2*	0.39	(0.25, 0.62)	0.49	(0.28, 0.85)
**Baby admitted to special care**						
No (ref)	379	*55.5*	1		n/s	
Yes	39	*48.2*	0.74	(0.47, 1.18)	n/s	
**Gestation**						
< 37 weeks (ref)	31	*50.0*	1		n/s	
37 weeks and over	387	*55.1*	1.12	(0.70, 1.79)	n/s	
**Breastfed within 1 hour of birth**						
Yes (ref)	246	*57.8*	1		n/s	
No	172	*50.9*	0.71	(0.54, 0.93)	n/s	
**Received formula in hospital**						
No (ref)	328	*60.0*	1		1	
Yes	90	*41.5*	0.39	(0.29, 0.53)	0.43	(0.30, 0.62)
**Education**						
Completed tertiary (ref)	269	*63.9*	1		n/s	
Completed secondary (but not tertiary)	96	*48.5*	0.53	(0.38, 0.75)	n/s	
Did not complete secondary	53	*36.6*	0.33	(0.22, 0.48)	n/s	
**Family income (AUD)**						
<$20,000 (ref)	43	*42.2*	1		n/s	
$20–50,000	149	*52.0*	1.43	(0.95, 2.12)	n/s	
>$50,000	206	*62.2*	2.06	(1.38, 3.09)	n/s	
Income missing	20	*45.5*	1.09	(0.59, 2.02)	n/s	
**Region of birth**						
Australia (ref)	269	*50.3*	1		1	
Asia	64	*75.3*	2.91	(1.84, 4.62)	2.90	(1.57, 5.36)
Other	85	*59.0*	1.58	(1.11, 2.24)	1.53	(0.99, 2.36)
**Employed/studying at 6 months**						
No (ref)	306	*57.2*	1		n/s	
Yes	112	*48.9*	0.75	(0.56, 0.99)	n/s	
**Attended childbirth education**						
Yes (ref)	364	*58.8*	1		1	
No	54	*37.2*	0.45	(0.32, 0.63)	0.46	(0.29, 0.71)
**Breastfeeding problems day 2–4**						
No (ref)	178	*57.6*	1		n/s	
Yes	240	*52.8*	0.73	(0.55, 0.96)	n/s	
**Rated midwife home visit extremely helpful for breastfeeding issues**						
No (ref)	321	*53.3*	1		n/s	
Yes	85	*60.7*	1.36	(0.93, 1.97)	n/s	
Did not have home visit	12	*54.6*	1.05	(0.45, 2.47)	n/s	
**Attended breastfeeding clinic**						
Had breastfeeding problems but did not attend clinic (ref)	177	*50.0*	1		n/s	
Had breastfeeding problems, did attend clinic	79	*55.6*	1.25	(0.85, 1.85)	n/s	
Had no bf problems	162	*60.5*	1.53	(1.11, 2.12)	n/s	
**Anxiety or depression problem in first 6 months (self-report)**						
No (ref)	280	*58.0*	1		1	
Yes	138	*49.1*	0.78	(0.59, 1.03)	0.64	(0.46, 0.90)
**Relationship problems at all in first 6 months**						
No (ref)	378	*57.0*	1		1	
Yes	40	*39.6*	0.56	(0.38, 0.84)	n/s	
**Age at recruitment (continuous variable used)#**	-	-	0.48 #	(0.35, 1.84)	1.58 #	(1.35, 1.86)

* Number of women who gave that response who were continuing to breastfeed at 6 months

** Proportion of women who gave that response who were continuing to breastfeed at 6 months

***Adj: adjusted; bf: breastfeeding; n/s: not significant, not retained in model

# ORs for age are the odds per five year increase in age

The factors that remained in the final model that were *positively *associated with breastfeeding any breast milk at six months were: a very strong desire to breastfeed (AdjOR 2.18, 95%CI 1.45, 3.29); having been breastfed oneself as a baby (AdjOR 1.73, 95% CI 1.19, 2.54); the woman being born in an Asian country (AdjOR 1.57, 95%CI 1.57, 5.36); and older maternal age (AdjOR per 5 year increase in age 1.58, 95% CI 1.35, 1.86). There was an increasing association with increasing age. Factors that were *negatively *associated with feeding any breast milk at six months were: a woman having no intention to breastfeed for six months or more (AdjOR 0.41, 95%CI 0.25, 0.67); the baby receiving formula while in hospital (AdjOR 0.43, 95%CI 0.30, 0.62); the mother smoking 20 or more cigarettes per day pre-pregnancy (AdjOR 0.47, 95%CI 0.26, 0.86); not attending childbirth education (AdjOR 0.46, 95%CI 0.29, 0.71); maternal obesity (AdjOR 0.49, 95%CI 0.28, 0.85); and self-reported anxiety or depression which was a problem in the six months after birth (AdjOR 0.64, 95%CI 1.35, 1.86).

## Discussion

The findings from this logistic regression analysis are similar to associations reported in other studies. Breastfeeding intentions/desire to breastfeed [5]; increasing maternal age [9,15,18,20,22,23]; and a history of having been breastfed oneself [40-42] were associated with longer duration of breastfeeding. Smoking status [19,21,23]; and non-attendance at childbirth education classes [9] were associated with shorter duration. The findings also add to the literature in other areas which have been less reported on, such as the association between breastfeeding outcomes and infants receiving formula in hospital, maternal obesity, and having self-reported depression or anxiety in the six months after the baby is born. The association found here between being Asian-born compared with Australian-born has not been reported in other studies; there appears to be no consistent pattern in breastfeeding outcomes based on ethnicity [19,22,31,32]. Although education is a factor consistently associated with breastfeeding outcomes [7,9,11,14,19,21,24-26], it did not remain significant in this analysis. For this group of women other factors had a greater impact.

Given the complexity of factors that affect breastfeeding outcomes, the question of 'where to from here?' is difficult to answer. Empirical evidence points to very few areas where interventions have an effect in a population such as the Australian one, which has relatively high breastfeeding initiation. There is a number of more vulnerable groups which have consistently been shown to be at increased risk of ceasing breastfeeding early or not commencing at all, such as younger mothers, those with less education, women who smoke, women not planning to breastfeed (or those who are unsure how they will feed their baby) and women who come from families where there has not been a culture of breastfeeding. One way of addressing these issues might be to choose to work with those groups, and test interventions that have been shown to be successful elsewhere. Likewise, implementing and evaluating interventions showing positive results, such as volunteer peer support for breastfeeding, would be an option. However, the context in which breastfeeding takes place *is *complex and multidimensional, so it may be that further work on the other aspects impacting on breastfeeding (such as difficulties breastfeeding away from home and/or in public places) should be explored concurrently with some of the measures mentioned here.

Antenatal breastfeeding intention is a strong indicator of breastfeeding initiation and duration [5], and this seems to be the case across almost all groups of women, such as those with less formal education, younger women and those with less social support. This an important area to focus on in future interventions aimed at increasing breastfeeding. Deciding to breastfeed prior to becoming pregnant compared with making a later decision has been associated with longer duration of breastfeeding [17,42,43], so interventions that aim to increase women's intention to breastfeed before pregnancy may also be of benefit. In this study women who had *either *an antenatal or postnatal intention to breastfeed six months or more were just as likely to be breastfeeding at six months as those who had this intention at both time points, suggesting that interventions during pregnancy that aim to increase women's intention to breastfeed may have an effect. It is important to also keep in mind that some of the variables that were included in the model may have some relationship to each other, for example it may be that some babies were not breastfed within one hour of birth, or received formula in hospital because the mother did not have such a strong intention to breastfeed. Some cross-over such as this is perhaps inevitable, but should be kept in mind, and associations checked prior to inclusion in models such as this.

It is likely that women's opinions of optimal breastfeeding duration, in Australia at least, may not match WHO recommendations, even among those who do breastfeed, and that health care providers' expectations of the optimum length of breastfeeding differ from women's views and expectations. Despite the recognised health benefits of breastfeeding for at least six months, it may be that the WHO goals, or the notion of maintaining breastfeeding to at least six months (regardless of whether the existence of the WHO goals is known), is viewed as neither desirable nor feasible in some populations.

The results regarding the associations between smoking and breastfeeding outcomes for the women who participated in ABFAB are consistent with the literature; however, this still needs to be interpreted in light of the fact that smoking itself is strongly associated with having no partner, having a lower income, being less educated, being depressed and being more likely to be exposed to violence [44]. Women who smoke *may be *less motivated or have less intention to breastfeed, [28,45] but again this may related to other contextual factors. In this study we used self reported pre-pregnancy smoking to include in our regression model, and the negative association found between smoking and breastfeeding was confined to women who reported smoking 20 or more cigarettes per day pre-pregnancy. Of those who reported smoking 20 or more per day pre-pregnancy, 68% (42/62) continued to smoke in pregnancy, compared to 31% (62/203) of those who reported smoking less than 20 pre-pregnancy.

Women were probably more likely to agree to participate in the study if they had an interest in breastfeeding, and only six women who participated in the trial responded at recruitment that they intended to formula feed. In the original trial context this applied equally to women in all three trial arms, and is a common issue in any prospective study. In terms of this analysis it could be viewed as positive. That is, the factors found to be associated with breastfeeding duration in this analysis pertain to a group of women who, on the whole, intended to breastfeed.

The study was limited to primiparous women who could speak, read and write English. A further restriction on the study population was that women choosing birth centre or private obstetric care were excluded from the outset. These factors may limit the generalisability of the study findings. We did not seek the views of partners and family directly.

## Conclusion

Factors *positively *associated with breastfeeding any breast milk at six months were: a very strong desire to breastfeed; having been breastfed oneself as a baby; the mother being born in an Asian country; and being older. Factors *negatively *associated with feeding any breast milk at six months were: a woman having no intention to breastfeed for six months or more; baby receiving formula while in hospital; smoking 20 or more cigarettes per day pre-pregnancy; not attending childbirth education; maternal obesity; and having self-reported anxiety or depression which was a problem in the six months after birth. Of these, the factors that have been less reported in the literature included: giving babies formula in hospital; maternal obesity; and self-reported maternal depression or anxiety in the six months after the baby is born. Interventions that seek to increase breastfeeding should consider focusing on women who are most at risk of early discontinuation of breastfeeding.

## Competing interests

The author(s) declare that they have no competing interests.

## Authors' contributions

DF was involved in study coordination and implementation, questionnaire design and primary data analysis, and drafted the manuscript. HM was involved in study coordination and implementation, questionnaire design and data analysis. JL was involved in overseeing study implementation, coordination, questionnaire design, and data analysis. All authors read and approved final manuscript.
